# Conversion back to bevacizumab or ranibizumab for recurrent neovascular activity with aflibercept in age-related macular degeneration: a case series

**DOI:** 10.1186/s40942-016-0028-9

**Published:** 2016-01-25

**Authors:** Geraldine R. Slean, Kornwipa Hemarat, Rahul N. Khurana, Jay M. Stewart

**Affiliations:** 1 Department of Ophthalmology, University of California, San Francisco, 10 Koret Way, K301, San Francisco, CA 94143-0730 USA; 2 Department of Ophthalmology, California Pacific Medical Center, 2340 Clay Street, 5th floor, San Francisco, CA 94115 USA; 3 Maharat Nakhon Ratchasima Hospital, Nakhon Ratchasima, Thailand; 4 Northern California Retina Vitreous Associates, 2485 Hospital Dr. #200, Mountain View, San Jose, CA 94040 USA

**Keywords:** Refractory age-related macular degeneration, Aflibercept, Bevacizumab, Ranibizumab

## Abstract

**Background:**

Neovascular age-related macular degeneration often requires chronic therapy with anti-VEGF agents, and patients with recurrent disease are challenging to manage.

**Methods:**

This retrospective case series evaluates patients who were switched from bevacizumab or ranibizumab to aflibercept and then back again because of recurrent fluid on optical coherence tomography (OCT) by reporting changes in OCT measurements over the course of medication changes.

**Results:**

Twenty-one eyes in nineteen patients received an average of 20.7 bevacizumab and/or ranibizumab injections and then an average of 7.2 aflibercept injections before being switched back to bevacizumab or ranibizumab because of recurrent fluid on OCT. Median central macular thickness improved on transition from bevacizumab or ranibizumab (317 μm) to aflibercept (285 μm; p = 0.034), then worsened over the course of aflibercept treatment (296 μm; p = 0.080), but improved again with transition from aflibercept back to bevacizumab or ranibizumab (283 μm; p = 0.016). The total volume of subretinal fluid, intraretinal fluid, and pigment epithelial detachments also decreased on transition from bevacizumab or ranibizumab (2.56 mm^3^) to aflibercept (2.44 mm^3^; p = 0.080), then worsened over the course of aflibercept treatment (3.18 mm^3^; p = 0.019), and improved again on transition back to bevacizumab or ranibizumab (2.11 mm^3^; p = 0.016).

**Conclusions:**

While aflibercept appears initially effective, some patients develop recurrent fluid with aflibercept that improves with transition back to bevacizumab or ranibizumab. Rotating anti-VEGF agents may be beneficial with recurrent neovascular activity.

## Background

Intravitreal anti-vascular endothelial growth factor (VEGF) medications bevacizumab and ranibizumab are recognized for improving visual outcomes and decreasing macular fluid in patients with neovascular age-related macular degeneration (AMD) [1, 2]. Over time, decreased responsiveness to these medications has been described in some patients [3–5]. Since late 2011, aflibercept has been shown to have similar improvements in visual outcomes and macular fluid as bevacizumab or ranibizumab [6]. Patients demonstrating recurrent or persistent fluid on bevacizumab or ranibizumab have been transitioned to aflibercept, with many patients responding favorably [7]. However, a select number of patients have worsened with persistent or recurrent fluid on aflibercept [8–10]. Having demonstrated persistent or recurrent fluid on bevacizumab or ranibizumab and aflibercept, these patients are a challenge to treat. We report a series of patients who initially responded well to aflibercept after having been treated with ranibizumab or bevacizumab but subsequently developed recurrences of fluid, and then were switched back to either ranibizumab or bevacizumab.

## Methods

Institutional review board approval was granted by the University of California, San Francisco (UCSF), and all research was compliant with the Health Insurance Portability and Accountability Act. This exploratory, retrospective case series examined UCSF and Northern California Retina Vitreous Associates (NCRVA) patients with neovascular AMD between January 1, 2012 and March 14, 2014 who had been initially treated with multiple injections of 0.5 mg ranibizumab or 1.25 mg bevacizumab, then switched to 2 mg injections of aflibercept due to persistent or recurrent fluid on optical coherence tomography (OCT), and subsequently switched back to 0.5 mg ranibizumab or 1.25 mg bevacizumab, again, due to recurrent or persistent fluid on OCT. Criteria for medication switch to aflibercept and then back to ranibizumab/bevacizumab were the same: the presence of persistent or recurrent macular fluid, namely intraretinal fluid or subretinal fluid. Treatment schedules were dependent on the discretion of the treating retinal specialist and patient availability. Patients were only included if they had received at least three injections of bevacizumab or ranibizumab prior to aflibercept, followed by at least three injections of aflibercept, in order to better evaluate response to these medications.

Outcome variables included best available visual acuity (VA) converted to logMAR (logarithm of minimum angle of resolution), central macular thickness (CMT) [9, 11], and estimated volumes of pigment epithelium detachments (PED), subretinal fluid (SRF), and intraretinal fluid (IRF) features when present on OCT (centered on the fovea) before and after medication changes from ranibizumab or bevacizumab to aflibercept and then from aflibercept to bevacizumab or ranibizumab. OCT 1 represents the last OCT taken while the patient was receiving ranibizumab or bevacizumab prior to aflibercept. OCT 2 represents the first OCT taken after being switched to aflibercept. OCT at time point 3 is the last OCT taken while the patient was on aflibercept, prior to being switched back to ranibizumab or bevacizumab. Finally, OCT at time point 4 represents the first OCT taken while the patient was retreated with ranibizumab or bevacizumab. VA was collected during the same visits as OCT images. OCT images were acquired with the Heidelberg Spectralis (Heidelberg Engineering, Carlsbad, California) at UCSF and the Cirrus (Carl Zeiss Meditec, Dublin, California) at NCRVA. Machines had tracking software to ensure that the same area in each eye was scanned on repeat visits. With Spectralis volume scans, the 19 B-scans were 240 μm apart; whereas with Cirrus volume scans, the 128 B-scans were 47 μm apart.

Since OCT-calculated segmentation lines demonstrate a degree of error, especially in cases of severe AMD [12], all measurements were obtained manually using the caliper tool. CMT was obtained by measuring the distance between the foveal depression (or expected foveal depression in severe cases of IRF) and Bruch’s membrane. Estimated volumes of macular fluid on OCT were calculated by adapting simplified volumetric grading [13]. The maximum width and height of a feature were measured and the number of B-scans on which the feature appeared were counted. These calculations were then multiplied by distance between B-scans, yielding an estimated volumetric cube encapsulating the feature.

Demographic data included patient age and sex, number of ranibizumab, bevacizumab, and aflibercept injections, and timing of each of those injections.

VA and OCT measurements were not normally distributed and are presented as medians with interquartile ranges. Changes in these variables over time (between sequential OCTs: OCT 1, OCT 2, OCT 3, OCT 4) were analyzed using Wilcoxon signed-rank tests. Friedman’s tests were performed for each of the variables (CMT, SRF, IRF, PED, Total Volume, VA) to assess for differences over the course of medication changes. Patients were also divided by treatment group: patients who had received ranibizumab, then aflibercept, and then ranibizumab again; patients who had received bevacizumab, then aflibercept, and then bevacizumab again; and patients who had received all three medications over the course of their treatment. Medians and interquartile ranges of OCT measurements were evaluated for each treatment group. Given the nature of our non-parametric data, we did not feel ANOVA was appropriate to evaluate differences between treatment groups. Instead, Wilcoxon signed-rank tests were again used to evaluate changes between sequential OCTs, and Friedman’s tests were performed for each of the OCT variables by treatment group. Statistical analyses were performed using Stata 14 (StataCorp, College Station, Texas).

## Results

At UCSF, 80 eyes with neovascular AMD were transitioned from bevacizumab or ranibizumab to aflibercept. Of these, 25 eyes (31 %) were transitioned back to ranibizumab after at least one aflibercept injection; 9 eyes (11.3 %) were transitioned off aflibercept after at least three injections. Patients were transitioned from aflibercept to ranibizumab after a single injection because of immediate worsening on OCT or patient preference given immediate worsening of VA. We identified 21 eyes in 19 patients at UCSF and NCRVA who met inclusion criteria (Table 1) and had received at least three ranibizumab or bevacizumab injections prior to aflibercept and then at least three aflibercept injections. These 21 eyes had received an average of 20.7 ranibizumab and/or bevacizumab injections (10 eyes had only received ranibizumab, 4 eyes had only received bevacizumab, and 7 eyes had had both), followed by an average of 7.2 aflibercept injections before being switched back to ranibizumab or bevacizumab (with 14 eyes transitioning to ranibizumab and 7 eyes transitioning to bevacizumab) by retinal specialists because of persistent or recurrent fluid on OCT. Patients received aflibercept injections every 6 weeks on average, and were then treated with ranibizumab or bevacizumab about 6 weeks after their last aflibercept injection. Thus, in this group of patients, aflibercept represented 2nd-line treatment in 14 eyes, and 3rd-line treatment in 7 eyes that had received sequential periods of ranibizumab as well as bevacizumab prior to aflibercept.

**Table 1:** Patient characteristics

Total eyes, N	21
Total patients, N	19
Female, N (%)	11 (58)
Age, years (range)	79 (67–95)
Number of bevacizumab and ranibizumab injections prior to aflibercept, mean (range)	20.71 (3–49)
Aflibercept injections, mean (range)	7.19 (3–14)
Injection interval in weeks while on aflibercept, mean ± SD	6.08 ± 1.12
Injection interval in weeks between last aflibercept injection and first bevacizumab or ranibizumab injection, mean ± SD	5.97 ± 3.24

Description of patients with chronic neovascular age-related macular degeneration transitioned from bevacizumab or ranibizumab to aflibercept and then back to bevacizumab or ranibizumab

**Fig. 1:**
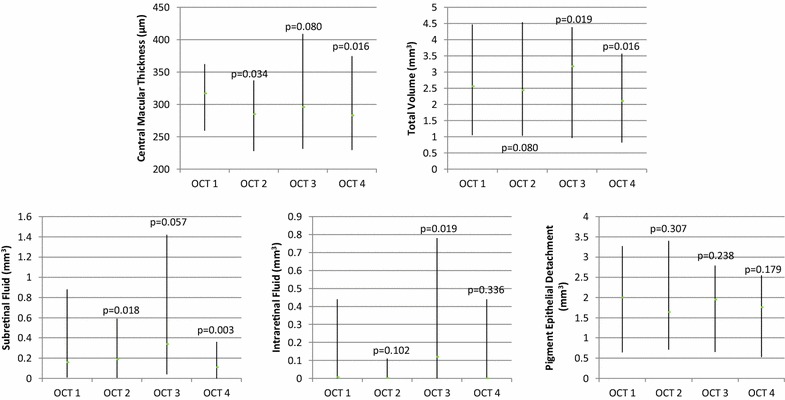
Optical coherence tomography (OCT) changes over the course of medication changes. Median values and interquartile ranges of central macular thickness, total fluid volume, subretinal fluid, intraretinal fluid, and pigment epithelial detachments over the course of anti-VEGF medication changes from OCT 1 (while patients were on bevacizumab or ranibizumab prior to aflibercept treatment) to OCT 2 (shortly after transitioning to aflibercept treatment), then OCT 3 (while patients were on aflibercept treatment prior to transition back to bevacizumab or ranibizumab), and finally OCT 4 (shortly after patients were re-started on bevacizumab or ranibizumab)

Median CMT improved from 317 µm (interquartile range (IQR), 259.5–362.5 μm) on ranibizumab or bevacizumab at OCT time point 1 to 285 µm (IQR, 228–337 μm) on aflibercept at OCT time point 2 (p = 0.034) (Table 2), then worsened to 296 µm (IQR, 231.5–409 μm; p = 0.080) at OCT time point 3 during aflibercept treatment, and improved again to 283 µm (IQR, 229.5–374.5 µm; p = 0.016) at OCT time point 4 when switched back to ranibizumab or bevacizumab. Of note, CMT on returning back to ranibizumab or bevacizumab (283 µm) was significantly improved from prior measurement while on ranibizumab or bevacizumab before aflibercept (317 µm; p = 0.029). A similar trend in macular fluid was noted over the course of medication changes. The total volume of SRF, IRF, and PED decreased from 2.56 mm^3^ (IQR, 1.05–4.47 mm^3^) on ranibizumab or bevacizumab to 2.44 mm^3^ on aflibercept (IQR, 1.03–4.54 mm^3^; p = 0.080), then increased during aflibercept treatment (3.18 mm^3^; IQR, 0.96–4.39 mm^3^; p = 0.019), and decreased again upon switching back to ranibizumab or bevacizumab (2.11 mm^3^; IQR, 0.82–3.57 mm^3^; p = 0.016). Median values for SRF, IRF, and PED showed a tendency to improve with the transition from ranibizumab or bevacizumab to aflibercept, then worsen over the course of aflibercept treatment, and improve again on transition from aflibercept back to ranibizumab or bevacizumab, but not all of these changes proved to be statistically significant on Wilcoxon signed-rank tests. In the majority of eyes, SRF improved significantly (p = 0.018) on transition to aflibercept from ranibizumab or bevaciumab even though the median showed a small increase from 0.16 to 0.19 mm^3^. SRF (p = 0.057) and IRF (p = 0.019) worsened during the course of aflibercept treatment, and SRF (p = 0.003) showed significant improvement on transition back to ranibizumab or bevacizumab from aflibercept. SRF on transition back to ranibizumab or bevacizumab was also significantly improved from prior measurement on ranibizumab or bevacizumab before aflibercept (p = 0.036) (Fig. 1). All other changes over time were non-significant. Percent change in OCT measurements between sequential OCT time points was <25 % for all measurements, except for a median 35 % decrease in SRF from OCT 1 to OCT 2, a 37 % decrease in SRF from OCT 3 to OCT 4, and a 26 % decrease in Total Volume from OCT 3 to OCT 4. Friedman’s tests for all variables (CMT, SRF, IRF, PED, total volume) were statistically significant (p < 0.001) and confirm differences in macular features over the course of multiple medication changes.

**Table 2:** Optical coherence tomography measurements and visual acuity over the course of medication changes

	Median (interquartile range)	Wilcoxon p value	Friedman’s p value
Central macular thickness (CMT), μm			<0.001
OCT 1	317 (259.5–362.5)		
OCT 2	285 (228–337)	0.034^a^	
OCT 3	296 (231.5–409)	0.080^b^	
OCT 4	283 (229.5–374.5)	0.016^c^	
Total fluid volume^d^, mm^3^			<0.001
OCT 1	2.56 (1.05–4.47)		
OCT 2	2.44 (1.03–4.54)	0.080^a^	
OCT 3	3.18 (0.96–4.39)	0.019^b^	
OCT 4	2.11 (0.82–3.57)	0.016^c^	
Subretinal fluid (SRF), mm^3^			<0.001
OCT 1	0.16 (0.01–0.88)		
OCT 2	0.19 (0.006–0.59)	0.018^a^	
OCT 3	0.34 (0.04–1.42)	0.057^b^	
OCT 4	0.11 (0–0.36)	0.003^c^	
Intraretinal fluid (IRF), mm^3^			<0.001
OCT 1	0.007 (0–0.44)		
OCT 2	0.002 (0–0.11)	0.102^a^	
OCT 3	0.12 (0–0.78)	0.019^b^	
OCT 4	0.0005 (0–0.44)	0.336^c^	
Pigment epithelial detachment (PED), mm^3^			<0.001
OCT 1	2.00 (0.64–3.27)		
OCT 2	1.64 (0.71–3.40)	0.307^a^	
OCT 3	1.95 (0.65–2.79)	0.238^b^	
OCT 4	1.76 (0.53–2.55)	0.179^c^	
Visual acuity (VA), logMAR^e^			<0.001
OCT 1	0.30 (0.18–0.48)		
OCT 2	0.30 (0.18–0.44)	0.175^a^	
OCT 3	0.30 (0.14–0.57)	0.292^b^	
OCT 4	0.30 (0.18–0.60)	0.104^c^	

Treatment response of patients with chronic neovascular age-related macular degeneration switched from bevacizumab or ranibizumab to aflibercept and then back to bevacizumab or ranibizumab

OCT 1 represents the last OCT on bevacizumab or ranibizumab prior to transition to aflibercept. OCT 2 represents the first OCT after switching to aflibercept. OCT 3 indicates the last OCT on aflibercept before transition back to bevacizumab or ranibizumab. OCT 4 represents the first OCT after switching back to bevacizumab or ranibizumab

^a^Comparing response from bevacizumab or ranibizumab (OCT 1) to aflibercept (OCT 2)

^b^Comparing progression while on aflibercept (OCT 2 and OCT 3)

^c^Comparing response back to bevacizumab or ranibizumab (OCT 4) from aflibercept (OCT 3)

^d^Summation of subretinal fluid, intraretinal fluid, and pigment epithelial detachments

^e^Logarithm of minimum angle of resolution

Only two patients received bevacizumab, then aflibercept, followed by a return to bevacizumab. Ten patients received ranibizumab followed by aflibercept before being transitioned back to ranibizumab. Nine patients received all three medications over the course of their treatment. Medians and interquartile ranges (IQR) of OCT measurements are presented for the latter two treatment groups (Table 3). Overall, patients who had received all three medications over the course of their treatment demonstrated less macular volume than patients in the ranibizumab-aflibercept-ranibizumab group. All Friedman’s tests for CMT, total volume, SRF, IRF, and PED by treatment group were statistically significant.

**Table 3:** Changes in optical coherence tomography measurements by treatment group

	Ranibizumab-aflibercept-ranibizumab treatment group (N = 10)	Wilcoxon p value	Friedman’s p value	Ranibizumab/bevacizumab-aflibercept-ranibizumab/bevacizumab treatment group (N = 9)	Wilcoxon p value	Friedman’s p value
	Median (interquartile range)			Median (interquartile range)		
Central macular thickness (CMT), μm			0.002			0.001
OCT 1	335.5 (308.25–381.75)			283 (252.5–382.5)		
OCT 2	302.5 (222–360.75)	0.030^a^		285 (220.5–442.5)	0.38^a^	
OCT 3	375 (292.75–435.25)	0.016^b^		295 (216.5–392.5)	0.22^b^	
OCT 4	314 (232.5–399.75)	0.021^c^		248 (212.5–333.5)	0.037^c^	
Total Fluid Volume^d^, mm^3^			0.007			0.003
OCT 1	3.50 (2.47–5.52)			1.68 (0.71–5.28)		
OCT 2	3.55 (1.49–4.73)	0.121^a^		1.36 (0.58–4.20)	0.221^a^	
OCT 3	4.28 (2.94–6.27)	0.018^b^		0.96 (0.76–3.85)	0.297^b^	
OCT 4	2.87 (2.31–4.31)	0.024^c^		0.77 (0.47–2.72)	0.087^c^	
Subretinal fluid (SRF), mm^3^			0.007			0.003
OCT 1	0.11 (0.013–1.12)			0.16 (0.003–0.82)		
OCT 2	0.15 (0–0.76)	0.091^a^		0.11 (0.006–0.59)	0.077^a^	
OCT 3	0.68 (0.01–1.68)	0.178^b^		0.33 (0.04–1.15)	0.077^b^	
OCT 4	0.06 (0–0.40)	0.004^c^		0.09 (0.008–0.31)	0.038^c^	
Intraretinal fluid (IRF), mm^3^			0.003			0.010
OCT 1	0.04 (0–1.74)			0 (0–0.73)		
OCT 2	0.008 (0–0.07)	0.032^a^		0.0002 (0–0.49)	0.426^a^	
OCT 3	0.22 (0.0006–0.96)	0.012^b^		0 (0–0.59)	0.335^b^	
OCT 4	0.0009 (0–0.99)	0.206^c^		0 (0–0.44)	0.377^c^	
Pigment Epithelial Detachment (PED), mm^3^			0.0003			0.003
OCT 1	2.39 (1.77–3.23)			0.67 (0.18–3.85)		
OCT 2	2.36 (1.35–4.38)	0.222^a^		0.83 (0.20–3.40)	0.430^a^	
OCT 3	2.49 (1.90–4.13)	0.070^b^		0.86 (0.18–2.63)	0.107^b^	
OCT 4	2.39 (2.05–3.23)	0.480^c^		0.50 (0.16–2.03)	0.187^c^	

Treatment response of chronic neovascular age-related macular degeneration patients by treatment group: ranibizumab-aflibercept-ranibizumab vs. ranibizumab/bevacizumab-aflibercept-ranibizumab/bevacizumab

Patients were divided into three treatment groups: those patients who had received bevacizumab only, then aflibercept, and then bevacizumab again (bevacizumab-aflibercept-bevacizumab), those patients who had received ranibizumab only, then aflibercept, and then ranibizumab again (ranibizumab-aflibercept-ranibizumab), and those patients who had received all three medications over the course of their treatment (ranibizumab/bevacizumab-aflibercept-ranibizumab/bevacizumab). The bevacizumab-aflibercept-bevacizumab group contained only two patients and was thus excluded from further analysis

^a^Comparing response from bevacizumab or ranibizumab (OCT 1) to aflibercept (OCT 2)

^b^Comparing progression while on aflibercept (OCT 2 and OCT 3)

^c^Comparing response back to bevacizumab or ranibizumab (OCT 4) from aflibercept (OCT 3)

^d^Summation of subretinal fluid, intraretinal fluid, and pigment epithelial detachments

Median VA showed little apparent change with transition from ranibizumab or bevacizumab (logMAR 0.30; IQR, 0.18–0.48) to aflibercept (logMAR 0.30; IQR, 0.18–0.44; p = 0.175), over the course of aflibercept treatment (0.30; IQR, 0.14–0.57; p = 0.292), or transition back to ranibizumab or bevacizumab (0.30; IQR, 0.18–0.60; p = 0.104). Friedman’s test was statistically significant over the course of medication changes.

No adverse events were recorded as a result of intravitreal injection.

## Discussion

Our retrospective study included patients with recurrent or increasing persistent fluid on OCT while on ranibizumab or bevacizumab who had been switched to aflibercept and then switched back to ranibizumab or bevacizumab because of persistent or recurrent fluid on OCT while on aflibercept. OCT measurements exhibited a unifying trend: CMT and macular fluid volume showed some improvement with initial transition from ranibizumab or bevacizumab to aflibercept, but these improvements dissipated over the course of aflibercept treatment. After switching back to bevacizumab or ranibizumab, CMT and macular volume once again showed some initial improvements. Moreover, CMT and SRF on transition from aflibercept to bevacizumab or ranibizumab were improved even beyond last measurement on bevacizumab or ranibizumab prior to starting aflibercept.

Evaluation of response to anti-VEGF medication is often reserved for at least 1 month after completion of loading doses (or 3 monthly doses) [14], but many patients show immediate worsening on aflibercept, likely due to innate resistance. The patients in this study were given at least three aflibercept injections, then monitored for response, and found to have worsening persistent or recurrent fluid. Poor or worsening response could represent decreased treatment effectiveness or insufficient dosing. Since the majority of these patients demonstrated a modest initial improvement on OCT (albeit not >25 % improvement in many measurements) in response to medication changes, one could argue that this decreased effectiveness constitutes poor response or tachyphylaxis, rather than innate resistance which is characterized by worsening response from the first injection [11]. Tachyphylaxis, a clinical phenomenon referring to a progressive decrease in therapeutic response after repetitive administration of a pharmacological active substance, has been well documented with numerous medications, including ranibizumab and bevacizumab [3–5]. While the longer dosing intervals in our case series do not allow us to evaluate for a possible role of tachyphylaxis, patients on consistent 4-week aflibercept dosing intervals who experience recurrent fluid on OCT may be suffering from a waning effect of aflibercept, or tachyphylaxis.

Improvement with transition to aflibercept may be explained by the medication’s increased binding affinity for vascular endothelial growth factor-A (VEGF-A), VEGF-B, and placental growth factor [11]. Multiple reports have demonstrated that patients with ranibizumab or bevacizumab tachyphylaxis respond positively to aflibercept [15]. Improvement with transition back to ranibizumab or bevacizumab is harder to explain but may be due to the different molecular structure of the medications. Ehlken et al. have shown that nonresponders to bevacizumab had VA improvements when switched to ranibizumab, or vice versa [16]. Gasperini et al. demonstrated reductions in macular fluid in 81 % of patients with choroidal neovascularization who had developed tachyphylaxis on ranibizumab or bevacizumab and were switched to the other anti-VEGF medication [32]. These studies suggest that patients may respond differently to all three anti-VEGF drugs. The mechanism of poor response is unclear but may be cellular, metabolic, and/or genetic. Poor response may involve compensatory signaling that leads to upregulation of pro-angiogenic factors and/or downregulation of anti-angiogenic factors, neutralizing antibodies, and/or genetic predisposition [14]. Moreover, many of the patients in this study have had AMD for many years and may also be suffering from changes in vessel wall structure or fibrosis that limit the effect of these medications [17].

Management of patients who demonstrate a poor response or non-response to multiple anti-VEGF agents is proving difficult. One suggested treatment option includes periodic transition between the various anti-VEGF agents [3, 11, 18]. In our study, CMT and macular fluid had somewhat positive, albeit temporary, responses to anti-VEGF medication switches. In particular, CMT and SRF responded to ranibizumab or bevacizumab even after these eyes had been previously deemed non-responsive to these medications, suggesting that drug rotation may help stabilize fluid accumulation. Medication vacation periods may be helpful [19]. Czarnowicki et al. [20] show that psoriasis plaques re-exposed to halobetasol ointment after a vacation period demonstrate greater improvement than plaques not previously exposed to halobetasol. Switching between different medications in the same class allows for an individual medication vacation period while at the same time continuing to treat a condition. Yang et al. [21] describe switching back and forth between intrathecal bupivacaine and lidocaine to achieve pain relief. In our study, initial improvements in CMT and macular fluid volume were immediately apparent after starting aflibercept and after re-starting ranibizumab or bevacizumab. Other studies have shown decreases in CMT and macular fluid on aflibercept for chronic AMD patients with persistent or recurrent fluid, even after one injection [10, 22]. Thus, periodic transition between the various anti-VEGF agents may be an effective treatment option.

The question arises as to when to transition between the various anti-VEGF agents. In their comprehensive literature review, Lazzeri et al. suggest that CMT decrease by <100 µm or lack of total fluid reabsorption after 3–6 injections of a single anti-VEGF agent (namely ranibizumab or bevacizumab) should call into question the drug’s effectiveness and warrant consideration of transition to aflibercept or another treatment option [11]. Amoaku et al. define a poor response to anti-VEGF treatment as <25 % decrease in central retinal thickness (CRT) and/or new or persistent IRF or SRF with minimal improvement in VA (0 to −4 ETDRS letters). They define non-response as unchanging CRT, SRF, IRF and/or PED with a worsening in VA (> −5 ETDRS letters) [14]. We feel that poor response or non-response to 3–6 monthly aflibercept injections warrants transition to another anti-VEGF agent or a change in therapeutic strategy.

Shorter treatment intervals may also prove beneficial in managing these patients. Biweekly dosing of ranibizumab or bevacizumab has proven beneficial in poorly responsive patients [23]. Future studies are needed to evaluate the usefulness of aflibercept injections given <28 days. Administration of aflibercept every 8 weeks may be too long for some patients, especially those with persistent fluid [24, 25]. The significant increase in total macular volume over the course of multiple aflibercept injections (mean 7; range 3–14) in our study may indicate the need for shorter time intervals between injections and/or an increasing non-responsiveness to aflibercept over time. Unfortunately, monthly (or even more frequent) dosing of anti-VEGF medications is not always achievable in the real world, but should be considered in these difficult to manage patients. Additional treatment possibilities include increased drug dosages [18, 26, 27] and combined treatment with other medications [3, 14]. Schaal et al. [28] report improved outcomes when combining triamcinolone acetate with bevacizumab. Patients demonstrating increasing non-responsiveness should also be evaluated for additional lesions or diagnoses with consideration of photodynamic therapy for polypoidal choroidal vasculopathy [29].

Prior evaluations of response to anti-VEGF medications on OCT have focused mostly on qualitative analyses, retinal thickness estimates [8, 22], or PED height [7, 11]. However, volumetric quantitative measurements provide greater insight into the response of SRF, IRF, and PED. Similar to other qualitative studies [3, 8, 21, 30], PEDs in our study showed comparatively less change in response to anti-VEGF medications as compared to other features on OCT. Also consistent with findings in other studies, VA did not change over the course of medication changes in these patients with chronic AMD [7, 9–11, 22], suggesting that irreversible damage has occurred to retinal structures [31] or other factors such as outer retinal morphology (external limiting membrane integrity, outer retinal layer thickness) [32–34] may play more important roles in VA than central macular fluid accumulations or central macular thickness.

Limitations of this study include small sample size, retrospective nature, lack of standardized VA measurements, several treating physicians acting without a standard treatment protocol, and short follow-up after return to ranibizumab or bevacizumab.

## Conclusions

In conclusion, recurrence of neovascular activity can occur in patients with AMD on aflibercept therapy. Although these patients showed moderate initial CMT and macular fluid improvement on aflibercept, improvements waned over the course of multiple aflibercept treatments, suggesting a need for shorter time intervals between injections. On transition back to ranibizumab or bevacizumab, patients again demonstrated initial improvement in CMT and macular fluid, sometimes even beyond last recorded measurements on ranibizumab or bevacizumab (prior to starting aflibercept). Since these patients showed some initial improvement on the different anti-VEGF medications, they may benefit from periodic rotation between the various anti-VEGF agents.
